# The Engagement in Physical Activity for Middle-Aged and Older Adults with Multiple Chronic Conditions: Findings from a Community Health Assessment

**DOI:** 10.1155/2013/152868

**Published:** 2013-09-05

**Authors:** Wei-Chen Lee, Marcia G. Ory

**Affiliations:** ^1^Health Policy & Management, Program on Health Workforce Analysis and Policy, School of Rural Public Health, Texas A&M Health Science Center, College Station, TX 77843-1266, USA; ^2^Health Promotion & Community Health Sciences, Program on Healthy Aging, School of Rural Public Health, Texas A&M Health Science Center, College Station, TX 77843-1266, USA

## Abstract

The current aging trends accompanying the increasing prevalence of multiple chronic conditions (MCCs) and decreasing participation in physical activity (PA) have swept the United States. In light of the magnitude of this phenomenon, this study seeks to identify the most common MCC combinations and their relationships with PA level. A cross-sectional study, *Brazos Valley Health Assessment*, was conducted between October 2009 and July 2010. All data analyses were performed by STATA 12.0. The overall sample which met the inclusion criteria is 2,603. Among people older than 45 years, chronic conditions of cardiovascular, endocrine, and musculoskeletal systems were the most prevalent. Participants with three chronic conditions were less likely to meet the PA standard than those with only two chronic conditions. Younger age, women, rural residence, and unsafe environments were related to the lower PA level. After adjusting for seven covariates, all MCCs combinations adversely affect the level of PA (OR < 1.0, *P* < 0.05). People with MCCs were among the least active subgroups despite the health benefits of doing exercise. Given the well-documented benefits of physical activity for delaying the onset or progression of MCCs, public health efforts to enhance regular PA in middle-aged and older adults are recommended.

## 1. Introduction

As seen globally [1], the population in the US is aging [2]. There were approximately 120.1 million adults aged 45 years or older (39.30% of the total US population) in 2011, and this number is expected to rise to 162.8 million (42.96% of the total US population) by 2030 [3]. The rapid escalation of the aging population has been accompanied by an increase in the prevalence of multiple chronic conditions (MCCs) [4, 5].

The recent national emphasis on MCCs (defined as two or more chronic conditions) can be attributed, in part, to studies documenting the association of MCC with increased risks of adverse health outcomes, higher health care use, and associated costs [6–8]. Recent national data reported that around 63 million Americans had MCCs, and this number is projected to grow to 81 million by 2020 [9, 10]. For people aged 45–64 years old, the percentage of adults with two or more of nine selected chronic conditions has increased 5 percent from 1999 to 2009 [11]. This number is more than 8 percent for the group of people older than 65 years of age. The prevalence of MCCs in Medicare or veteran populations has been further explored. Two-thirds of Medicare beneficiaries (around 21.4 million) had at least two or more chronic conditions which were associated with more hospitalizations, doctor visits, home health services, ER visits, and hospital readmissions [6]. Also their average Medicare spending is higher than the spending of beneficiaries with less than one chronic condition. The mean number of chronic conditions (out of 23 selected conditions) in veterans aged 65 and older was found to be 5.5 for men and 5.1 for women [12]. Both hypertension and hyperlipidemia were presented in the 9 of the 15 most prevalent triplets (i.e., a combination of three conditions). 

Concurrently, the lack of physical activity (PA) is well recognized as a risk factor for chronic diseases [13]. It is likely that even more Americans will fail to meet national PA guidelines (e.g., being active for at least 30 minutes of moderate intensive activity for most days of the week) in the decades to come as the population ages [14, 15]. While PA is important for health promotion and disease prevention across individual's life course, older adults have less engagement in PA than younger age groups [16–18]. The percentages of adults participating in PA that met the national physical activity guidelines were 19.2% among people aged 45–54 years, 15.9% 55–64 years, 13.6% 65–74 years, and 7.3% 75–84 years, but only 4.0% among people aged 85 years and older [19]. Findings from these national statistics conclude that older people have lower levels of PA. The latest data from Behavioral Risk Factor Surveillance System (BRFSS) also noted that the percentage of no leisure-time physical activity (LTPA) is the highest in the oldest age group as compared to the younger age groups [20]. Multilevel policies are recommended to promote physical activity [7].

The provision of the new American health care reform law, the Patient Protection and Affordable Care Act (PPACA), supports the development and implementation of new approaches to help patients manage multiple chronic conditions [21]. Insufficient PA has been identified as a major risk factor for the onset and progression of multiple chronic conditions [22]. But the level of PA conducted by people with MCCs has not been specifically studied. The majority of prior studies focus on the relationship of single chronic condition such as diabetes or chronic obstructive pulmonary disease (COPD) and levels of PA in comparison to those without any chronic conditions [23, 24]. 

Only a few empirical studies address the relationships between MCCs and the engagement in or barriers to PA. For example, symptoms of one of chronic conditions or lifestyle behaviors that interfered with another condition/behavior often impede the population with MCCs from doing exercise [25]. One participant could not exercise for diabetes due to the difficult breathing, a syndrome of the asthma. One qualitative study suggests that long-term limitations on activity accompanying their MCC were the strongest barrier to PA participation [26]. These long-term physical symptoms consisted of pain/discomfort, fatigue/dizziness, weakness/lack of strength, shortness of breath, and poor balance. Other barriers found include poor-to-average self-rated general health, lack of social support, and transportation issues [27]. 

None of previous studies identify which combination of chronic conditions contributes the most to reduced engagement in PA. Further, there has been relatively little research conducted on MCCs which are grounded in a person-based perspective rather than a disease-based perspective. Previous studies primarily regard multiple chronic conditions or syndromes as a whole or a single concept. Nevertheless, different combinations of MCC should have different impacts on a person's quality of care and quality of life. Therefore, this study examines how the top three prevalent combinations affect the PA level of middle-aged and older adults. 

There is a critical need to address the relationships between MCCs and involvement in PA and to identify whether there is a specific pattern of MCCs that is associated with different amounts PA involvement, controlling for other known correlates of PA. To address the current dearth of information in this area, three research objectives were proposed to (1) explore the prevalence of all types of MCC, (2) analyze the relationships between the top three prevalent combinations of MCC with the PA level and personal characteristics of middle-aged and older adults, and (3) examine the impacts of MCC on the PA level with controlling personal characteristics. 

## 2. Methods

### 2.1. Data Source

This cross-sectional study draws on data from the 2010 Brazos Valley Health Status Assessment (BVHSA), a joint effort of the Brazos Valley Health Partnership (BVHP) and the Center for Community Health Development (CCHD) at the Texas A&M Health Science Center [28]. The survey was conducted between October 2009 and July 2010 in the Brazos Valley and one adjacent county, an area where more than 300,000 citizens live within 5,109 square miles [29]. The final version of the assessment also included other existing data collected by the Texas Department of State Health Services (DSHS), the 2000 Census, the CDC, the Texas Workforce Commission, and two projects *Healthy People 2010* as well as *Rural Healthy People 2010* [28].

### 2.2. Survey Design and Study Sample

From a comprehensive list of residential addresses, 15,000 households were randomly selected, and 10,501 were reached by phone [28]. Residents willing to participate in the survey received the survey instrument primarily in English, instructions, and a self-addressed stamped envelope. Overall, 5,362 agreed to complete a survey, and 3,360 households returned a complete survey (62.6%). For purpose of this study, we focused on participants who were at least 45 years old and who had at least one chronic condition. The data of 2,603 residents meeting the inclusion criteria was analyzed with the approval of Institutional Review Board at the Texas A&M University.

### 2.3. Dependent Variable: Level of Physical Activity (PA)

In the survey, physical activities were categorized into moderate- and vigorous-intensity types. Descriptions of moderate activity (e.g., brisk walking) and vigorous activities (e.g., running) were provided to help respondents anchor their responses. The research participants were asked how many days per week they did moderate and/or vigorous activities for at least 10 minutes at a time. They were also asked how much total time per day they spent doing these activities. Through multiplying both answers (i.e., days per week and minutes per day), we obtained the total minutes of physical activities they participated in every week. The 2008 Physical Activity Guidelines for Americans recommends adults to have at least 150 minutes of moderate-intensity aerobic activity or at least 75 minutes of vigorous-intensity aerobic activity every week [22]. According to this guideline, the research participants were divided into two groups: one group who met the standard and another who failed to meet the PA national standard for adults.

### 2.4. Independent Variables: Combinations of Chronic Conditions

A chronic condition is a condition that requires ongoing medical services and is slow in its progress and long in its continuance [30, 31]. The survey participants were asked about whether they have been told by a medical care provider that they had any of the 21 selected conditions by circling *yes* or *no*. The 21 conditions could be classified into (1) central nervous system (e.g., depression, anxiety, developmental or learning disability, Parkinson's disease, and other mental health issues), (2) cardiovascular system (e.g., hypertension, congestive heart failure, high cholesterol, angina, heart attack, and stroke), (3) respiratory system (e.g., asthma and emphysema/chronic bronchitis/COPD), (4) endocrine system (e.g., obesity/overweight, diabetes, skin cancer, and nonskin cancer), (5) musculoskeletal disorders (e.g., arthritis/rheumatism), (6) genitourinary disorders (e.g., a disease of liver/cirrhosis/chronic hepatitis), and (7) gastro intestinal tract (e.g., inflammatory bowel disease/ulcerative colitis and stomach ulcer). 

This study defines MCC as a health status in which patients experience symptoms of two or more systems of chronic systems (e.g., the combination of central nervous system and cardiovascular system) rather than counting up all the specific cardiovascular related conditions separately. In other words, this approach takes a system rather than a disease-specific approach. The numbers and percentages of all combinations were estimated among the research participants aged 45 years or older. Also this study addressed the characteristics and PA involvement of the top three combinations, including the combination of cardiovascular and endocrine system (*n* = 293), the combination of cardiovascular and musculoskeletal systems (*n* = 143), and the combination of cardiovascular, endocrine, and musculoskeletal systems (*n* = 239). These three combinations were defined to be exclusive to each other and hence reflect three different groups of participants.

### 2.5. Covariates

Characteristics of participants and environmental factors are covariates in the analysis model. Questions about personal characteristics and environmental factors were asked and employed as covariates in the regression models. Self-reported personal characteristics include age (i.e., 45~64 or above 65 years old), gender (i.e., male or female), the highest grade of education (i.e., less than high school, high school, or higher than high school), race/ethnicity (i.e., non-Hispanic White, African American, Hispanic, or other races such as Asian, Black, or Native American), and residence (i.e., rural or urban areas). Participants were also asked about the accessibility and safety to engage in PA in the neighborhood defined as the area all around their homes that they could walk to in 10–15 minutes. Accessibility refers to three questions about whether there were sidewalks, shops/stores, and recreation facilities in the neighborhood. Safety refers to three questions about whether participants themselves or their neighbors could feel free to do activities or walk outside. A scale was constructed giving one point for each “yes” response. A higher total score indicates more walkable and activity friendly neighborhood.

### 2.6. Analysis

Descriptive statistics were used to describe each study variable and reflect the first research objective. A correlation matrix was performed between two independent variables to test for multicollinearity. Bivariate analyses were conducted to respond to the second research objective, examining the associations between the PA level and personal characteristics, environmental factors, and combinations of chronic conditions. To achieve the third research objective, the logistic regression model was used to explore the associations between disease combinations and the PA level adjusting to seven covariates. Results were expressed as  *β*  coefficients with their *P* values and 95% confidence intervals. Two-tailed *P* values less than or equal to 0.05 were considered statistically significant. All statistical analyses were performed using the STATA version 12. 

## 3. Results

### 3.1. Selection of Top 3 Prevalent MCCs

Overall, there were 2,603 adults aged 45 years or older. Multiple chronic conditions refer to a health status with chronic illnesses of two or more systems. Any two of 21 diseases selected in this survey could form 210 combinations, while any three of 21 diseases could form 1,330 combinations and so on. Among the combinations of two chronic conditions, the most prevalent one combined chronic problems is the cardiovascular and endocrine systems (*n* = 293, 11.26%, assigned as the group 1), followed by a combination of cardiovascular and musculoskeletal systems (*n* = 143, 5.49%, assigned as the group 2). Among the combinations of three chronic conditions, the most prevalent one is the combination of the cardiovascular, endocrine, and musculoskeletal systems (*n* = 239, 9.18%, assigned as the group 3). Other combinations do not account for more than 5% of the total participants, and so this study further analyzed participants' level of PA, demographics, and environmental factors only in those three groups.

### 3.2. Description of Participants in Total and in Different Type of MCC

A basic frequency distribution confirmed the completeness of our data with no variable having more than 5% of missing values (Table 1). The total number in Table 1 refers to all participants meeting the age inclusion criteria other than the three defined MCC groups. As indicated in Table 1, around half of our participants (*n* = 1,300) met the PA standard, but another half did not (*n* = 1,303). The group 3, representing those with three conditions, was least likely to meet the PA standard (46.44%). Overall, 45.02% of research sample are older than 65 years, 68.92% are women, 55.86% have received education more than the high school level, 80.95% are non-Hispanic White, 68.42% are living in rural areas, 51.25% have accessibility to facilities such as a gym, and 71.30% reported that it is safe to engage in physical activity in their neighborhoods. Compared to the distributions of individual characteristics among three MCC groups, those in groups 2 and 3 had higher proportions of persons who were older than 65 (from group 1 to 3 are 41.64%, 73.43%, and 66.11%). Next, there were higher proportions of male participants in the groups 1 and 3 than those in the group 2 (40.27%, 27.27%, 42.68%).  The percentages associated with education level, residence, accessibility, and safety are relatively similar across all groups.  The majority of respondents across all categories were non-Hispanic White (86.01%, 78.32%, and 74.48%). Hispanic populations did not account for more than ten percent in any of three groups.

### 3.3. Description of Participants Met or Not Met the PA Standard

For the level of PA (i.e., met or not met the standard), statistically significant variation (*P* < 0.05) across personal characteristics, environmental factors, and combinations of chronic conditions were shown in Table 2. The level of physical activity tends to be lower (i.e., not met) among people who are 45 to 64 years old (*χ* ^2^ = 5.48, *P* = 0.019), female (*χ* ^2^ = 53.02, *P* = 0.000), perceived their neighborhood to be unsafe (*χ* ^2^ = 4.00, *P* = 0.046), and report chronic conditions in the three systems (*χ* ^2^ = 10.34, *P* = 0.016). 

### 3.4. Relationships between PA Level and Each Type of MCC

Table 3 shows the relationship between the level of PA and three MCC combinations (group 1: cardiovascular and endocrine systems; group 2: cardiovascular and musculoskeletal systems; group 3: cardiovascular, endocrine, and musculoskeletal systems) using people who had zero or only one chronic condition to be the reference group. Controlling for seven covariates, people with any type of MCC had lower level of PA involvement (Odds Ratio = 0.721 for the group 1, 0.664 for the group 2, and 0.515 for the group 3; *P* < 0.05). The odds of meeting the PA standard were 0.721 times for people in the group 1 compared to the odds for those having zero or only one chronic condition. Similarly, the odds of meeting the PA standard for the groups 2 and 3 were smaller than for the people without multiple chronic conditions.

## 4. Discussion

With age, the percentages of people ever diagnosed with chronic diseases are increasing [20]. As in previous studies [32], chronic problems with respect to the musculoskeletal system (e.g., arthritis), cardiovascular system (e.g., hypertension), and endocrine system (e.g., diabetes) are the most prevalent found in this community assessment of middle-aged and older adults. Conditions related to the cardiovascular system were part of all three MCC groupings reflecting their especially high rates among our survey respondents. Public awareness campaigns and lifestyle interventions for lifetime risks of cardiovascular diseases such as smoking cessation, healthy diets, and active living are warranted to reduce the magnitude of cardiovascular conditions [33]. 

Simultaneously, the current aging process is accompanied by significant declines in the participation of physical activity [34]. This study finding is consistent with the report of a national survey, Behavioral Risk Factor Surveillance System (BRFSS), that only half of people older than 45 years have participated in 150 minutes or more of aerobic physical activity per week [20]. 

In this study, percentages of meeting the PA standard were lowest for people with a combination of conditions in three different systems. Warshaw in 2006 also reported that the activity limitations increase with the number of chronic conditions [35]. Not surprising, there are higher percentages of people older than 65 in the groups 2 and 3 which both include the chronic problem of musculoskeletal system confirming the well-cited age association with musculoskeletal problems [36]. On the other hand, there are higher percentages of male participants in the groups 1 and 3 which both have the chronic problem of the endocrine system. This finding is in line with another literature that male adults are more vulnerable to diabetes than their female counterparts [37]. Future research is recommended to explore how age and gender, interacting with over covariates, affect the onset and progression of musculoskeletal and endocrine conditions.

The policy implication (increase physical activity involvement) of this study is one of the focuses in the PPACA which encourages the development and implementation of interventions to care for older adults with MCC [5, 35]. Next, the findings of this study are consistent with the literature that female gender, rural residence, and no safe environments to do exercise were the risk factors of meeting the PA standard [14, 38–40]. However, people older than 65 years in this survey are more likely to meet the PA standard than middle-aged adults which is contrary to the findings of previous studies [19, 20, 41] but might be a reflection that older adults have more time for leisure activity. Education level, race, and accessibility do not show significant association with the level of PA. This might be due to the different demographic distribution of this Community Health Assessment from other national survey (e.g., this assessment represented more rural areas). 

This research adds to the scant knowledge on the relationship between three MCC combinations and the PA participation among middle-aged and older adults. This study suggests that chronic conditions might impede people from participating in physical activity regardless of a person's age, gender, education, race, and residence, as well as accessibility and safety of the neighborhood. One study provides a good example that women with chronic diseases were less active despite the health benefits of doing exercise [38]. 

The research results related to a seven-county region in Central Texas and may not allow for generalizability to all US populations. Additionally, the self-reported responses on both physical activity and chronic conditions might be subject to bias relative to more objective measures such as physician reports. Given these limitations, longitudinal work must also be conducted to determine the causal association between the MCCs and PA level using more objective measures. More efforts are also needed to develop and tailor appropriate PA programs to people with multiple chronic conditions [42].

## 5. Conclusions

In the face of rapidly escalating aging populations along with age-related declines in physical, cognitive, and/or social functions, regular physical activity has been recognized as one of the most important factors impacting the aging process [14, 43]. Over the past two decades, medications prescribed to prevent the complications or treat the symptoms of chronic illnesses have increased rapidly [35]. Multiple chronic conditions that lead to the loss of independence, quality of life, and economic costs will continue to strain the US healthcare system [5, 9]. The findings of this Community Health Assessment reported the three most common MCC combinations and levels of physical activity for respondents in each of these groupings. To support the goal of successful aging [44, 45], endeavors to help middle-aged and older adults maintain regular physical activity are critical. Meanwhile, additional interdisciplinary research including medical, social, health behavioral, and environmental areas is needed to advance this field.

## Figures and Tables

**Table 1:** Distribution of physical activity, demographics, and environmental factors.

Number (%)	Total population (*N* = 2,603)	Cardiovascular + endocrine (*N* = 293)	Cardiovascular + musculoskeletal (*N* = 143)	Cardiovascular + endocrine + musculoskeletal (*N* = 239)
Physical activity				
Not met	1,303 (50.06)	142 (48.46)	68 (47.55)	128 (53.56)
Met the standard	1,300 (49.94)	151 (51.54)	75 (52.45)	111 (46.44)
Personal characteristics				
(1) Age				
45–64 years old	1,431 (54.98)	171 (58.36)	38 (26.57)	81 (33.89)
≥65	1,172 (45.02)	122 (41.64)	105 (73.43)	158 (66.11)
(2) Gender				
Female	1,794 (68.92)	175 (59.73)	104 (72.73)	137 (57.32)
Male	809 (31.08)	118 (40.27)	39 (27.27)	102 (42.68)
(3) Education				
Less than high school	130 (4.99)	8 (2.73)	6 (4.20)	14 (5.86)
High school	993 (38.15)	97 (33.11)	64 (44.76)	88 (36.82)
More than high school	1,454 (55.86)	187 (63.82)	71 (49.65)	134 (56.07)
Missing	26 (1.00)	1 (0.34)	2 (1.40)	3 (1.26)
(4) Race				
Non-Hispanic White	2,107 (80.95)	252 (86.01)	112 (78.32)	178 (74.48)
African American	252 (9.68)	14 (4.78)	25 (16.68)	39 (16.32)
Hispanic	172 (6.61)	22 (7.51)	5 (3.50)	13 (5.44)
Others	63 (2.42)	5 (1.73)	3 (2.10)	7 (2.93)
Missing	9 (0.35)	0 (0.00)	0 (0.00)	2 (0.68)
(5) Residence				
Urban	805 (30.93)	96 (32.77)	42 (29.37)	78 (32.64)
Rural	1,781 (68.42)	195 (66.56)	100 (69.93)	160 (66.95)
Missing	17 (0.65)	2 (0.68)	1 (0.70)	1 (0.42)
Environmental factors				
(6) Accessibility of PA location				
No access	1,269 (48.75)	142 (48.46)	69 (48.25)	117 (48.95)
Have access	1,334 (51.25)	151 (51.54)	74 (51.75)	122 (51.05)
(7) Safety of PA environment				
Not safe	747 (28.70)	88 (30.03)	37 (25.87)	62 (25.94)
Safe	1,856 (71.30)	208 (69.97)	106 (74.13)	177 (74.06)

**Table 2:** Association between the level of physical activity and personal characteristics, environmental factors, and combinations of chronic conditions.

*N* (%)	Not met	Met the standard	*χ* ^2^ (*P* value)
Age			5.48 (0.019)
45–64	746 (52.13)	685 (47.87)	
65+	557 (47.53)	615 (52.47)	
Sex			53.02 (0.000)
Male	319 (39.43)	490 (60.57)	
Female	984 (54.85)	810 (45.15)	
Education			1.14 (0.566)
<High school	71 (54.62)	59 (45.38)	
High school	493 (49.65)	500 (50.35)	
>High school	729 (50.14)	725 (49.86)	
Race			0.87 (0.8333)
Non-Hispanic White	1,059 (50.26)	1,048 (49.74)	
African American	127 (50.40)	125 (49.60)	
Hispanic	85 (49.42)	87 (50.58)	
Others	28 (44.44)	35 (55.56)	
Residence			3.45 (0.063)
Rural	425 (52.80)	380 (47.20)	
Urban	870 (48.85)	911 (51.15)	
Accessibility			0.47 (0.492)
Unavailable	644 (50.75)	625 (49.25)	
Accessible	659 (49.40)	675 (50.60)	
Safety			4.00 (0.046)
Unsafe	397 (53.15)	350 (46.85)	
Safe	906 (48.81)	950 (51.19)	
MCC			10.34 (0.016)
0-1 disease	244 (41.85)	339 (58.15)	
Cardiovascular + endocrine	142 (48.46)	151 (51.54)	
Cardiovascular + musculoskeletal	68 (47.55)	75 (52.45)	
Cardiovascular + endocrine + musculoskeletal	128 (53.56)	111 (46.44)	

**Table 3:** Logistic regression to identify relationships between PA level and disease combinations, adjusted by covariates.

(Met standard versus not met)	O.R. (95% C.I.)	*P* value
Independent variable		
0 or 1 chronic disease (reference group)	1.00	
** **Cardiovascular + endocrine	0.721 (0.536, 0.970)	0.031
Cardiovascular + musculoskeletal	0.664 (0.444, 0.993)	0.046
Cardiovascular + endocrine + musculoskeletal	0.515 (0.368, 0.721)	0.000
Covariates		
(1) Age (65 years or older)	1.820 (1.408, 2.352)	0.000
(2) Gender (female)	0.524 (0.409, 0.673)	0.000
(3) Education		
High school	1.652 (0.842, 3.240)	0.145
More than high school	1.462 (0.747, 2.861)	0.267
(4) Race		
Non-Hispanic White	0.987 (0.651, 1.496)	0.951
African American	1.448 (0.865, 2.423)	0.159
Hispanic	0.952 (0.441, 2.056)	0.901
(5) Residence (rural)	1.377 (1.047, 1.811)	0.022
(6) Accessibility of PA location (accessible)	1.274 (0.973, 1.669)	0.078
(7) Safety of PA environment (safe)	1.296 (0.963, 1.744)	0.087
